# Development of Novel Antimicrobial and Antiviral Green Synthesized Silver Nanocomposites for the Visual Detection of Fe^3+^ Ions

**DOI:** 10.3390/nano11082076

**Published:** 2021-08-16

**Authors:** Azam Ali, Fiaz Hussain, Safira Attacha, Ambreen Kalsoom, Waseem Akhtar Qureshi, Muhammad Shakeel, Jiri Militky, Blanka Tomkova, Dana Kremenakova

**Affiliations:** 1 Department of Material and Textile Engineering, Technical University of Liberec, 46117 Liberec, Czech Republic; jiri.militky@tul.cz (J.M.); blanka.tomkova@tul.cz (B.T.); dana.kremenakova@tul.cz (D.K.); 2 Institute of Advanced Materials, Bahauddin Zakariya University, Multan 60000, Pakistan; fiazravian@gmail.com (F.H.); engr.shakeel3@gmail.com (M.S.); 3 Institute of Biotechnology and Genetic Engineering, The University of Agriculture Peshawar, Peshawar 25130, Pakistan; safira@aup.edu.pk; 4 Department of Physics, The Government Sadiq College Women University, Bahawalpur 63100, Pakistan; kalsoom.ambreen@gscwu.edu.pk; 5 Cholistan Institute of Desert Studies, Bagdad ul Jadeed Campus, The Islamia University of Bahawalpur, Bahawalpur 63100, Pakistan

**Keywords:** green synthesis, poly(tannic acid), silver nanocomposites, antibacterial, visual detection of ferric, antiviral activity, antifungal

## Abstract

In the current research, we present a single-step, one-pot, room temperature green synthesis approach for the development of functional poly(tannic acid)-based silver nanocomposites. Silver nanocomposites were synthesized using only tannic acid (plant polyphenol) as a reducing and capping agent. At room temperature and under mildly alkaline conditions, tannic acid reduces the silver salt into nanoparticles. Tannic acid undergoes oxidation and self-polymerization before the encapsulating of the synthesized silver nanoparticle and forms silver nanocomposites with a thick capping layer of poly(tannic acid). No organic solvents, special instruments, or toxic chemicals were used during the synthesis process. The results for the silver nanocomposites prepared under optimum conditions confirmed the successful synthesis of nearly spherical and fine nanocomposites (10.61 ± 1.55 nm) with a thick capping layer of poly(tannic acid) (~3 nm). With these nanocomposites, iron could be detected without any special instrument or technique. It was also demonstrated that, in the presence of Fe^3+^ ions (visual detection limit ~20 μM), nanocomposites aggregated using the coordination chemistry and exhibited visible color change. Ultraviolet-visible (UV–vis) and scanning electron microscopy (SEM) analysis also confirmed the formation of aggregate after the addition of the analyte in the detection system (colored nanocomposites). The unique analytic performance, simplicity, and ease of synthesis of the developed functional nanocomposites make them suitable for large-scale applications, especially in the fields of medical, sensing, and environmental monitoring. For the medical application, it is shown that synthesized nanocomposites can strongly inhibit the growth of *Escherichia coli* and *Staphylococcus aureus*. Furthermore, the particles also exhibit very good antifungal and antiviral activity.

## 1. Introduction

Heavy metal ions have attracted considerable attention among the research community because they play a pivotal role in many biological systems and have an extreme impact on the environment [1,2,3]. Among the various important elements that play a vital role in the survival of living organisms, iron is considered an essential component for many biological systems [4]. Iron is an essential component of many cellular processes such as deoxyribonucleic acid (DNA) synthesis, oxygen transportation, and cellular metabolism [5,6]. Iron levels directly influence human health, as its deficiency or overdose may lead to serious diseases such as amimia, cancer, anorexia, liver damage, and many age-related diseases [6,7,8,9]. Thus, many efforts have been made to develop innovative and accurate techniques such as atomic absorbance spectroscopy, fluorescent, electrochemical analysis, high-performance liquid chromatography, and magnetic resonance imaging for iron detection [10,11,12,13]. However, these complex techniques require very expensive measuring instruments, sample preparation, and a highly trained operator for sample analysis. Thus, it has been a great challenge to develop practical sensitive sensors for the detection of iron. Polymer-based sensors for the visual detection of ferric ions have been proven to be a promising alternative to conventional techniques due to their ease of synthesis, simplicity, high sensitivity, and selectivity [14,15,16].

Polymer-coated metallic nanocomposites have gained considerable attention due to their unique applications, especially in the field of catalysis [17,18], medical [19,20], energy storage devices [21], flexible electronics [22,23,24,25], and sensing [26]. However, current methods are multi-step, and they require high energy input for the fabrication of metallic nanocomposites [27]. Generally, nanocomposites are prepared in two steps: (1) development of core; and (2) subsequent shell formation [28,29]. Here, we assume that plant polyphenol-coated silver nanocomposites can be developed using a single-step green synthesis approach without using any crosslinker or high energy input.

One of the promising plant polyphenols is tannic acid (TA). It undergoes oxidative self-polymerization before coating on a metallic surface [30,31]. Furthermore, it has the tendency to chelate metal ions as well as to stabilize the nanoparticles at room temperature [32,33]. This uniqueness makes TA an ideal candidate for the synthesis of metal nanocomposites. However, current methods for the fabrication of metal-PTA nanocomposites with a shell of poly(tannic acid) (PTA) either require high energy input [31] or rely on Fe^3+^ ions as a coordinator between tannic acid to form a thick shell [34]. In this work, we explain that poly(tannic acid)-coated silver nanocomposites (Ag-PTA) can be developed at room temperature without using any crosslinker or high energy input. The synthesized silver nanocomposites were further used as a sensor for the visual and optical detection of Fe^3+^ ions and as an antibacterial agent. We developed a facile, highly selective, and sensitive method for the visual detection of Fe^3+^ ions. This visual detection technique depends on the aggregation phenomenon of synthesized silver nanocomposites and Fe^3+^ ions in the solution. Fe^3+^ ions coordinate with three galloyls groups of tannic acid, present on the surface of silver nanocomposites, from a stable and crosslinked structure which results in the aggregation of silver nanocomposites. The characteristic UV–vis absorbance peak (441 nm) and yellow color of the aqueous dispersions of silver nanocomposites dispersions disappear. The color change can be observed by the naked eye. It was reported that encapsulated silver nanocomposites with higher aqueous stability have a strong effect against pathogens (viruses, bacteria, and fungus) [17,35]. Thus, we analyzed the antibacterial, antiviral, and antifungal behavior of the synthesized silver nanocomposites by incubating them with bacteria (*Escherichia coli* and *Staphylococcus aureus), viruses* (*Influenza virus* and *Feline calicivirus)* and yeast (Candida albicans). Results confirmed that synthesized nanocomposites have good antibacterial activity, antiviral, and antifungal activity with low cell toxicity. Based on their unique sensing and good behavior against pathogens, the synthesized silver nanocomposites may find a strong position among other metallic nanocomposites.

## 2. Experimental Section

### 2.1. Chemicals

Silver nitrate (AgNO_3_), tannic acid (C_76_H_52_O_46_), sodium hydroxide (NaOH), sodium borohydride (NaBH_4_), trisodium citrate (Na_3_C_6_H_5_O_7_), and ferric chloride hexahydrate (FeCl_3_⋅6H_2_O), were purchased from Sigma-Aldrich. Potassium chloride (KCl), sodium chloride (NaCl), and magnesium sulfate anhydrous (MgSO_4_) were obtained from Merck (Berlin, Germany). Rubidium chloride (RbCl), copper(II) sulfate pentahydrate (CuSO_4_⋅5H_2_O), calcium chloride (CaCl_2_), manganese chloride tetrahydrate (MnCl_2_⋅4H_2_O), nickel sulfate hexahydrate (Ni_2_SO_4_⋅6H_2_O), cadmium chloride (CdCl_2_), cobalt(II) nitrate hexahydrate (Co(NO_3_)_2_⋅6H_2_O), aluminum potassium sulfate dodecahydrate (AlK(SO_4_)_2_.12H2O), potassium chromium(III) sulfate dodecahydrate (KCr(SO_4_)_2_⋅12H_2_O), and neodymium(III) chloride hexahydrate (NdCl_3_⋅6H_2_O) were purchased from Sigma-Aldrich. All the chemicals were stored in the desiccator and used without any further purification. Deionized water (DI) was used in all the synthesis processes.

### 2.2. Synthesis of Silver Nanocomposites

**Figure 1:**
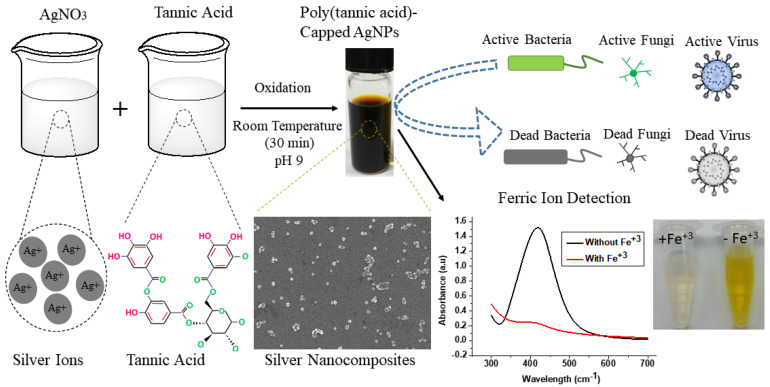
Schematic for the preparation of (core-shell) silver nanocomposites.

In a typical experiment, solutions of tannic acid (1 mM), AgNO_3_ salt (0.147 M), and NaOH (0.2 M) were prepared separately using distilled water at room temperature. The homogeneous mixture of AgNO_3_ salt (10 mL) and tannic acid (35 mL) was prepared at room temperature by magnetic stirring (500 rpm) for 5 min. The pH of the solution was maintained at approximately 9 using NaOH (1.35 mL). The reaction mixture was simply transferred into the glass beaker. It was then subjected to magnetic stirring for 30 min at room temperature. Finally, the product was obtained by centrifugal separation (10,000 rpm for 15 min) and washed with distilled water thrice to remove unwanted chemicals. The freshly prepared solutions of all the reagents were used every time. The schematic for the preparation of core–shell silver nanocomposites is shown in Figure 1. The prepared concentrated solution was coated on cotton fabric by drop-coating method. The plain woven (150 GSM) cotton fabric (round shape and 1.5 cm in diameter) was selected. Subsequently, fabric was dried in vacuum oven at 60 °C for 1 h. Five different concentrations (0.5, 0.75, 0.1, 1.25, and 1.5 mg) of silver nanocomposites were applied.

### 2.3. Characterization

UV–vis absorption spectra of the synthesized silver nanocomposites were carried out using a spectrophotometer (Model: JASCO V-770). A high-power thin-film X-ray diffractometer (D8 Advance) was used to determine the crystal structure of the silver nanocomposites. The sample was prepared on silicon wafer substrate and X-ray diffraction (XRD) measurements were determined using Cu-K𝛼 radiation at a wide range of Bragg angles 2θ and at a scanning speed of 2θ/min. Schottky field emission scanning electron microscopy (FE-SEM, Model: JEOL JSM-7600F) was used to analyze the size distribution and surface morphology of the developed nanocomposites. A high-resolution transmission electron microscopy (HR-TEM, Model: JEM-3010) working at 200 kV was used to analyze the product at high magnification. For transmission electron microscopy (TEM) analysis, the product was dispersed in the DI water and drop coated on the carbon copper grids. The elemental analysis of the product was analyzed using an energy dispersive spectrometer (EDS, Model: X-MAX 50). Zetasizer Malvern Instrument (Model: Nano-SZ) was used to determine the zeta-potential analysis of the aqueous sample. An X-ray photoelectron spectrometer (XPS, Model: ESCALAB250) was used to analyze the composition of the product. A Fourier transform infrared (FTIR) spectrophotometer (JASCO, FT/IR-4700) was used to further analyze the chemical structure of the product. For FTIR analysis, a powder sample of the product was prepared, and the spectrum was obtained in the range of 300–4000 cm^−1^ at a 4 mm/min scanning speed.

### 2.4. Antibacterial Testing

The antibacterial effectiveness of all samples was detected by AATCC 147 (zone of inhibition test). This is a type of qualitative test method in which bacterial strains of Gram-positive *Staphylococcus aureus* (CCM 3953) and Gram-negative *Escherichia coli* (CCM 3954) were selected. Bacterial colonies of both strains were grown in the Luria–Bertani (LB) agar plates. Round-shaped fabric samples (1.5 × 1.5 cm^2^) containing various contents of silver nanocomposites (0.5, 0.75, 0.1, 1.25, and 1.5 mg) were prepared by drop coating. Three samples of each fabric sample along with reference (control, C, without any silver) were placed in the LB agar plates. The LB agar plates were placed in the incubator at standard conditions (37 °C, 24 h) and the zone of inhibition was determined.

### 2.5. Antifungal Activity Assessments

The antifungal properties of the treated cotton fabric were evaluated according to American Association of Textile Chemist and Colorists (AATCC) test method 100–2004. The diploid fungi Candida albicans (NCPF 3153) was used. Antifungal activities were expressed in terms of the percentage reduction in the microorganisms and calculated by Equation (1):

(1) $$\mathrm{Percentage}\;\mathrm{reduction}\;R\%=\frac{\left(A-B\right)}{A}\times 100$$

where *A* and *B* are the number of microorganisms colonies on untreated and treated fabrics, respectively.

### 2.6. Antiviral Activity Assessments

Antiviral activity was detected and checked according to ISO 18184. It is a quantitative test method to access the antiviral ability of textile. Types of viruses and testing conditions selected for the test are given in Table 1. Types of host cells and viruses (I. CrFK -the Crandell–Rees feline kidney cell (CRFK) is an immortalized cell line derived from the feline kidney that is utilized for the growth of certain vaccinal viruses [36]; II. Madin–Darby canine kidney (MDCK) cells are the most widely used cell line for the isolation and propagation of human influenza viruses [37]; III. Influenza A virus subtype H3N2 (A/H3N2) is a subtype of viruses that causes influenza (flu) [38]; IV. feline calicivirus (FCV) is a highly contagious virus that causes a mild to severe respiratory infection and oral disease in cats [39], and the testing conditions selected for the test are given in Table 1.

#### 2.6.1. TCID_50_ Method and Preparation of Growth Medium

TCID_50_ method was selected to check the dilution of the virus suspension that induces a CPE (cytopathic effect) in 50% of the cell unit. For this purpose, 96-well microplates were selected with other necessary sterilized apparatus.

The growth medium was made according to instruction ISO 18184 by using recipe (Grade 3 water, Kanamycin sulfate, Eagle’s essentials, sodium bicarbonate, inactivated fetal bovine serum).

The cryopreserved host cells were defrosted. Then, a new flask of cell culture was prepared (20 mL of growth medium). Subsequent host cells were transferred into flask. Then, flask was put into CO_2_ incubator at 37 °C for 24 h to culture the cells at the bottom of the flask. Subsequently, the cultured cells were converted to subcultures by using the recipe and process described in ISO 18184.

Twenty milliliters (20 mL) of growth medium was taken into container and added 1 mL of subculture cells. Then, 0.1 mL of solution was added into each well among the 96 wells of the microplates. These plates were then put into the incubator at 37 °C for 5 days. After 5 days, the multiplication of cells was confirmed by using a microscope. Subsequently, the surface of the cultured cells was washed by using the maintenance medium given in Table 1. This solution in flask was termed Flask A.

#### 2.6.2. Preparation of Viruses

##### Influenza Virus

The cryopreserved influenza virus was defrosted by repeating the same procedure as that adopted for the host cells. The defrosted influenza virus was transferred to a new test tube and started to dilute with maintenance medium. Hence, the concentration of the virus was adjusted to TCID_50_/mL. The 1 mL of adjusted influenza virus was inoculated on the surface of cells (in flask A) and spread to whole surface. Now, the flask was put back into the CO_2_ incubator at 34 °C for 1 h to absorb the virus into the cell. After 1 h, we added 20 mL of the maintenance medium and 30 µL of trypsin and returned the flask to the incubator at 37 °C for 1–3 days to multiply the influenza virus. After 3 days, the multiplied viruses were transferred to a centrifugal tube and centrifuged at 9 °C at 1000× *g* for 15 min. The supernatant suspension was taken from the centrifugal tube (influenza virus suspension), placed in the test tubes, and cryopreserved at −80 °C. We verified that the concentration of the virus was more than TCID_50_/mL.

##### Feline Calicivirus

The cryopreserved calicivirus was defrosted by repeating the same procedure as adopted for the influenza virus and host cell. The defrosted calicivirus was transferred to a new test tube and started to dilute with the maintenance medium. Hence, the concentration of virus was adjusted to TCID_50_/mL. The 1 mL of adjusted calicivirus was inoculated on the surface of cells (in flask A) and spread to whole surface. Now, the flask was put back into the CO_2_ incubator at 34 °C for 1 h to absorb the virus into the cell. After 1 h, 20 mL of maintenance medium and 30 µl of trypsin were added and again the flask was returned to the incubator at 37 °C for 1–3 days to multiply the calicivirus. After 3 days, the multiplied viruses were transferred into the centrifugal tube and centrifuged at 9 °C at 1000× *g* for 15 min. The supernatant suspension was taken from the centrifugal tube (calicivirus suspension), placed in the test tubes, and cryopreserved at −80 °C. We verified that the concentration of the virus was more than TCID_50_/mL.

#### 2.6.3. Principle

A sample with the dimensions of 20 mm × 20 mm was cut (but the overall mass should be 0.40 g and can be adjusted with extra/less material if required). There should be a total of 9 control pieces and 6 treated pieces. The 3 pieces of each substrate were used to test the effect of the fabric on the cells without virus (cytotoxicity), and 3 control pieces were used to recover the starting titer of virus. The remaining pieces were inoculated with 200 μl of virus at a concentration of ~107 TCID_50_ (giving a final concentration of 10^5^) and left for the contact time. Following the contact time, the fabric was recovered in 20 mL of cell culture media and enumerated onto an appropriate cell line. TCID_50_ was calculated following the appropriate incubation time. Antiviral activity was calculated by comparison of the antiviral test material to that immediate recovered from the control fabric. The pathogens (bacteria, virus, and yeast) used in this study are list in Table 2.

### 2.7. Detection of Fe^3+^ Ion

For the visual and optical detection of the Fe^3+^ ion, the aqueous solution of Fe^3+^ was mixed with the aqueous solutions of silver nanocomposites (1 mL) to prepare the final concentration in the range of 1.0–25 μM. Under the same conditions, we also analyzed the selectivity of the synthesized silver nanocomposites for Fe^3+^ ions using other monovalent, divalent, or trivalent ions instead of Fe^3+^ ions, including K^1+^, Na^1+^, Rb^1+^, Ag^1+^, Mg^2+^, Cu^2+^, Mn^2+^, Ni^2+^, Cd^2+^, Co^2+^, Al^3+^, Cr^3+^, and Nd^3+^, at a final concentration of 25 μM. The characteristic yellow color of the silver nanocomposites disappeared, indicating the successful detection of metal ions. The addition of Fe^3+^ ions resulted in the aggregation of silver nanocomposites. This aggregation of silver nanocomposites was due to the interaction between the metallic ions and nanocomposites, which was further investigated by the UV–vis spectra.

## 3. Results and Discussion

### 3.1. Synthesis of Silver Nanocomposites

**Figure 2:**
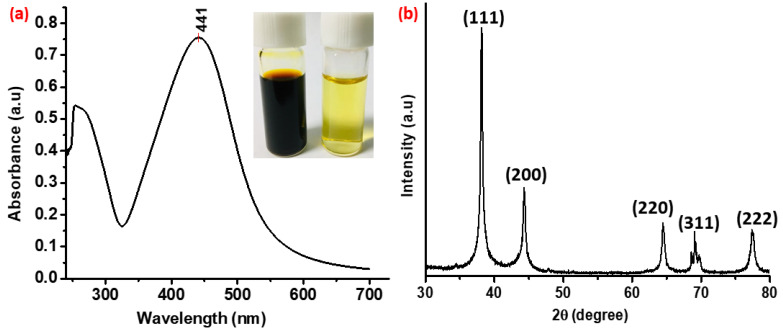
(**a**) UV–visible spectrum of the nanoparticle dispersion. The inset is the corresponding optical images of concentrated (~2300 ppm, left) and diluted (~50 ppm, right) aqueous dispersions of silver nanocomposites; and (**b**) the XRD pattern of highly crystalline synthesized silver nanocomposites.

The synthesis of silver nanocomposites was confirmed by the UV–visible spectrophotometer spectrum. The UV–vis spectrum of the diluted sample was determined in the range of 240–700 nm and the result is shown in Figure 2a. The aqueous dispersion of synthesized silver nanocomposites showed a strong absorbance band at 441 nm, which was a typical surface plasmon resonance band of silver nanocomposites. The peak observed at approximately 260 nm is attributed to the tannic acid after oxidation [40], indicating that synthesized nanocomposites are having a thick capping layer of poly(tannic acid). Furthermore, the synthesis of pure silver nanocrystals was confirmed by the X-ray diffraction (XRD) analysis. As shown in Figure 2b, there are five intense peaks in the XRD pattern which can be attributed to the bulk silver. The characteristics peaks observed at 38.2°, 44.3°, 64.5°, 69.3°, and 77.5° can be attributed to the (111), (200), (220), (311), and (222) planes of pure silver, respectively. The peak at 38.2 is the most intense among other peaks, indicating that the (111) plane is the most prominent in the crystal structure of silver nanocomposites.

**Figure 3:**
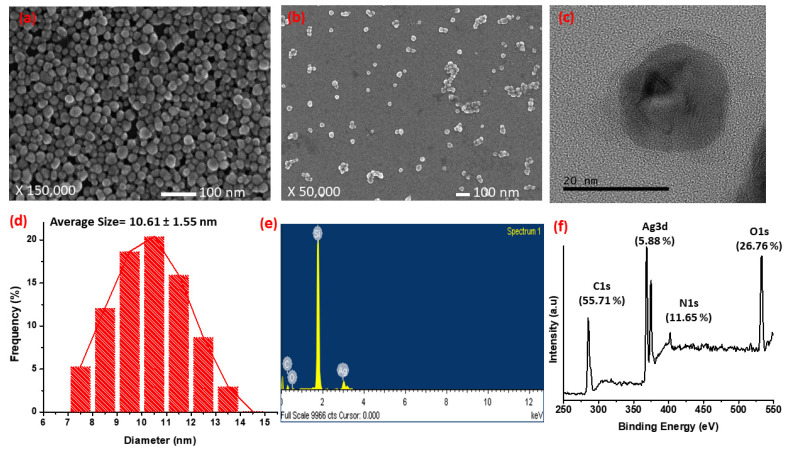
SEM image of concentrated (~2300 ppm (**a**) and diluted (~50 ppm) (**b**) silver nanocomposites; (**c**) TEM images of nanocomposites at higher magnification; (**d**) the size distribution analysis of as-synthesized silver nanocomposites, determined by ImageJ; (**e**) EDS pattern of synthesized silver nanocomposites; and (**f**) XPS pattern of the synthesized silver nanocomposites.

The surface morphology of silver nanocomposites was analyzed by the FE-SEM and HR-TEM. SEM images indicate that silver nanocomposites synthesized under optimum conditions are nearly spherical in nature with good mono-dispersity (Figure 3a,b). Additionally, the TEM image (Figure 3c) also confirms the synthesis of spherical silver nanocomposites encapsulated with a clear shell of poly(tannic acid) (approximately 3 nm). The size distribution shown in Figure 3d, calculated by using the ImageJ software from the SEM image, indicates that the synthesized silver nanocomposites have a narrow size distribution in the range of 7–15 nm with an average particle size of 10.61 ± 1.54. To determine the precise elemental composition of the synthesized silver nanocomposites, EDS analysis was performed, and the corresponding results are shown in Figure 3e. The synthesized nanocomposites contain silver (Ag), carbon (C), and oxygen (O) as the main chemical components. The strong peak observed at approximately 1.8 keV comes from the silicon wafer used as a substrate for sample preparation. The peaks observed at approximately 3 keV are due to the surface plasmon of silver while the other peaks at approximately 0.6 and 0.8 keV are attributed to the capping agent poly(tannic acid) (PTA). Thus, it can be concluded that in the synthesized silver nanocomposites, Ag is encapsulated by the thick layer of poly(tannic acid), as indicated by the TEM image (Figure 3c). Furthermore, the chemical composition of the product was determined by the XPS analysis (Figure 3f) and the results are summarized in Table 3. Fei et al. reported that C and O are the chemical constituents of the poly(tannic acid). While a small amount of nitrogen is attributed to some types of protein from plant polyphenols, the XPS results show that there is a very small amount of Ag in the nanocomposites, indicating that Ag exists as a core because XPS detects samples with limited thickness. Moreover, the binding energy peaks of O, C, and N in the synthesized silver nanocomposites shift from their positions compared to the pure tannic acid due to the oxidation of tannic acid.

**Figure 4:**
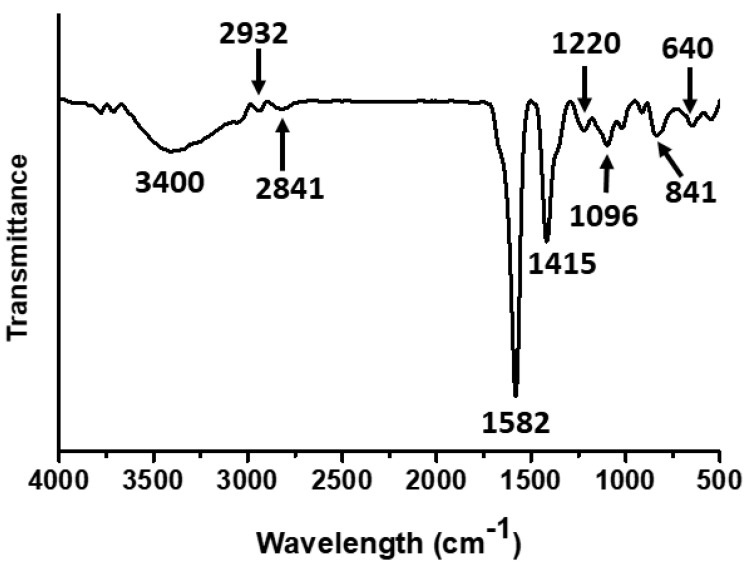
FTIR spectrum of synthesized silver nanocomposites.

The chemical structure of the synthesized silver nanocomposites was further investigated by FTIR analysis (Figure 4). The broad band at 3400 cm^−1^ was attributed to the -OH stretching of carboxylic acid. The bands at 2932 and 2841 cm^−1^ can be attributed to the CH- asymmetric and symmetric stretching, respectively, occurring in the poly(tannic acid). The strong absorbance band observed at 1582 cm^−1^ could be attributed to the C=O stretching of the carboxylic acid functional group. The absorbance band at 1415 cm^−1^ can be attributed to the C–C vibrations of aromatic rings, and the absorbance bands observed at 1220 cm^−1^ can be attributed to the ether bonds present in the synthesized silver nanocomposites. The band at 1096 cm^−1^ can be attributed to the C–O stretching. Furthermore, the absorbance bands in the range of 500–850 cm^−1^ are due to aromatic ring moieties of silver nanocomposites. For clarity and ease of understanding, the FTIR results are also summarized in Table 4. FTIR results confirm that silver nanocomposites were capped with oxidized tannic acid. Similar research findings were also reported by the previous researchers [31,35]. It is important to note that all our results are compatible with one another, indicating the successful synthesis of silver nanocomposites with a thick layer of poly(tannic acid).

### 3.2. Antibacterial Testing

**Figure 5:**
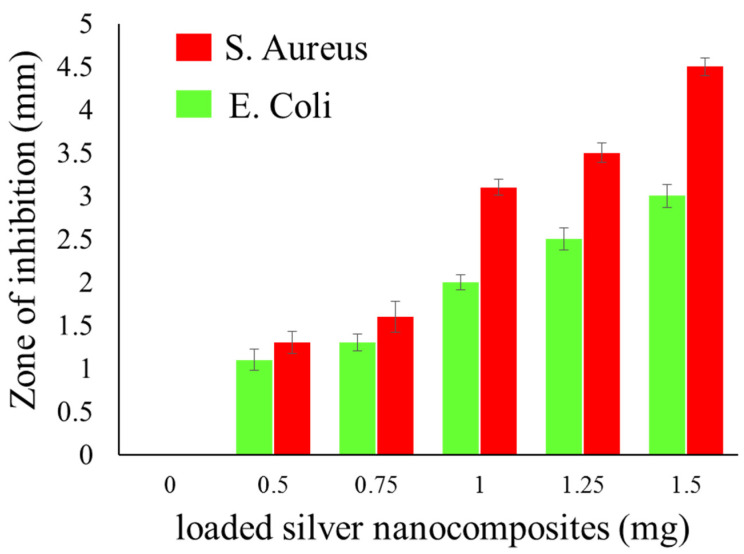
Summary of the antibacterial analysis of synthesized silver nanocomposites.

**Figure 6:**
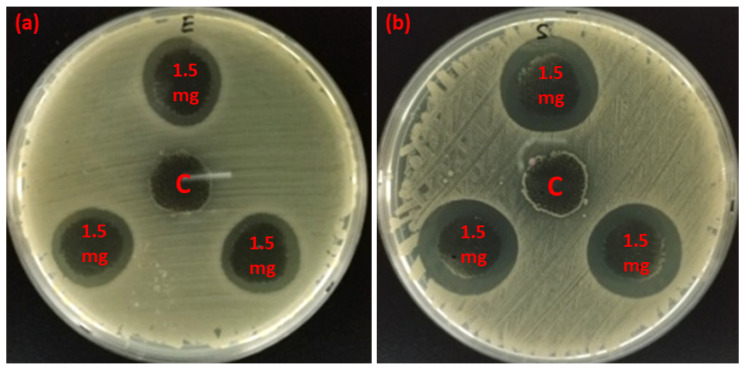
Zone of inhibition (ZOI) of sample E (containing 1.50 mg of synthesized silver nanocomposites) and control (C, containing 0.0 mg of synthesized silver nanocomposites) measured against (**a**) *E. coli* and (**b**) S. aureus microbial stains.

The qualitative standard test method was used to evaluate the antibacterial effectiveness of synthesized silver nanocomposite-coated fabrics. The control (C) samples and samples loaded with 0.5, 0.75, 0.1, 1.25, and 1.5 mg of silver nanocomposites were selected. The presence of a clear zone of inhibition around all treated samples confirmed the antibacterial activity. Results of antimicrobial activity tests are summarized in Figure 5. These results signify that an increase in the concentration of silver nanocomposites results in more nanosilver adsorbed on the fibers, causing an increase in the ZOI diameter. Moreover, for clarity and ease of understanding, the ZOI images against *E. coli* (Figure 6a) and *S. aureus* (Figure 6b) were also added. A clear zone of inhibition was shown around all the treated samples with 1.5 mg of silver nanocomposites.

The antimicrobial activity of silver is normally due to some common reason, as (1) silver effects on structural protective proteins of bacteria, (2) disrupt the cell wall and disturb the osmotic balance, (3) a source of oxidative stress to generate the hydrogen peroxide H_2_O_2_ and results in a type of Fenton reaction where precipitates of silver are formed. Eventually, this causes cell death [41,42,43]. The antibacterial activity of synthesized silver nanocomposite is higher for *S. aureus* (ZOI = 4.6 mm) compared to *E. coli* (ZOI = 3.2 mm). It is important to note that our results are compatible with the previously reported research [42,43].

In the case of *S. aureus*, the Ag ion inhibits the peptidoglycan (PGN) elongation due to the inactivation of PGN transglycosylase or transpeptidase (TP) and the enhancement of activation of PGN autolysins of amidases. However, in the case of *E. coli*, where the cell wall is comprised of lipopolysaccharide (LPS), lipoproteins (LP), and peptidoglycan (PGN) as the thinner layer within the periplasmic space. The Ag ions inhibit the biosynthesis of lipopolysaccharide due to active hydrolases, the destruction of the outer membrane structure by degrading of lipoprotein at C- and N-terminals, due to the inhibition of PGN formations by the inactivation of carboxypeptidase and TP-endopeptidase, and the activities of PGN autolysins of amidase, peptidase and carboxypeptidase [44,45]. Moreover, the antibacterial efficacy of green-synthesized silver nanoparticles more than that of silver ions, as AgNPs have a capping of phytochemicals that shows better reactivity to the bacterial protective layer due to possessing the adsorptive and biological activity with a different functional group of protein.

### 3.3. Antifungal and Antiviral Activity of Treated Samples

**Figure 7:**
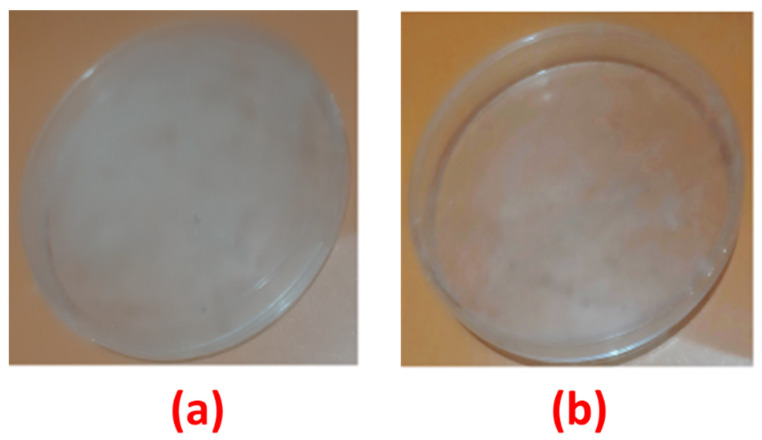
The effect of antifungal treatment on fabric samples: (**a**) sample without treatment; and (**b**) loaded with 1.5 mg of silver nanocomposites.

The qualitative standard test method was used to evaluate the antifungal effectiveness of synthesized silver nanocomposite-coated fabrics. The control (C) samples and samples loaded with 1.5 mg of silver nanocomposites were selected. The percentage reduction of fungi (*C. albicans*) with raw cotton and samples loaded with 1.5 mg of silver nanocomposites is presented in Table 5. The raw cotton fabrics without any antifungal agent can provide a suitable environment for microorganisms to grow, as shown in Figure 7.

**Figure 8:**
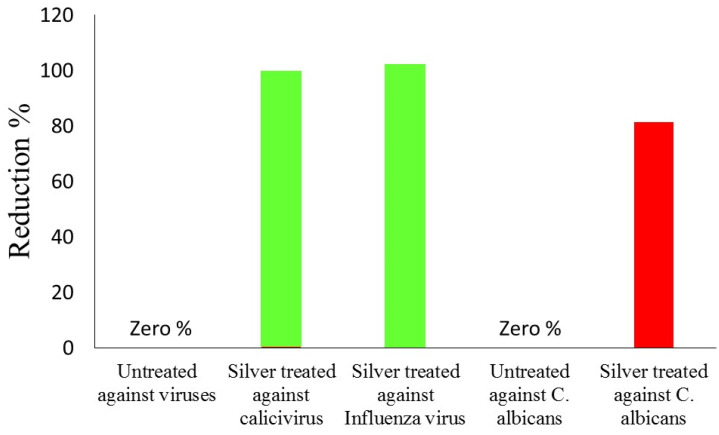
The reduction in virus infectivity in percentage against untreated sample and silver treated samples.

The quantitative standard test method was used to evaluate the antiviral effectiveness of synthesized silver nanocomposite-coated fabrics and the results are shown in Figure 8. The control (C) samples and samples loaded with 1.5 mg of silver nanocomposites were selected. Previous studies showed that silver nanoparticles showed antiviral effectiveness against the influenza A virus [46,47]. The theory explained that the antiviral activity of silver nanoparticles against several other types of viruses was due to the direct binding of the silver nanoparticles to viral envelope glycoproteins, thereby inhibiting viral penetration into the host cell, although the mechanism of action has not been well investigated [48,49]. The effect of particle size on the antiviral activity was usually observed, suggesting the spatial restriction of binding between virions and Ag NPs [50]. No antiviral activity was observed with cotton alone, showing that the antiviral activity of the composites was due to the bound silver nanocomposites. The treated fabric was the most efficacious against the feline calicivirus and influenza virus, achieving a reduction of 99.45% (2.19 log10) and 99.52% (2.30 log10) given two hours of exposure relative to the control fabric, as shown in Table 6. The effect of the size of the silver nanoparticles on antiviral ability suggests that influenza A virus selectively interacted with smaller Ag NPs, as previously reported for other types of viruses [51].

### 3.4. Visual Detection of Ferric Ions

**Figure 9:**
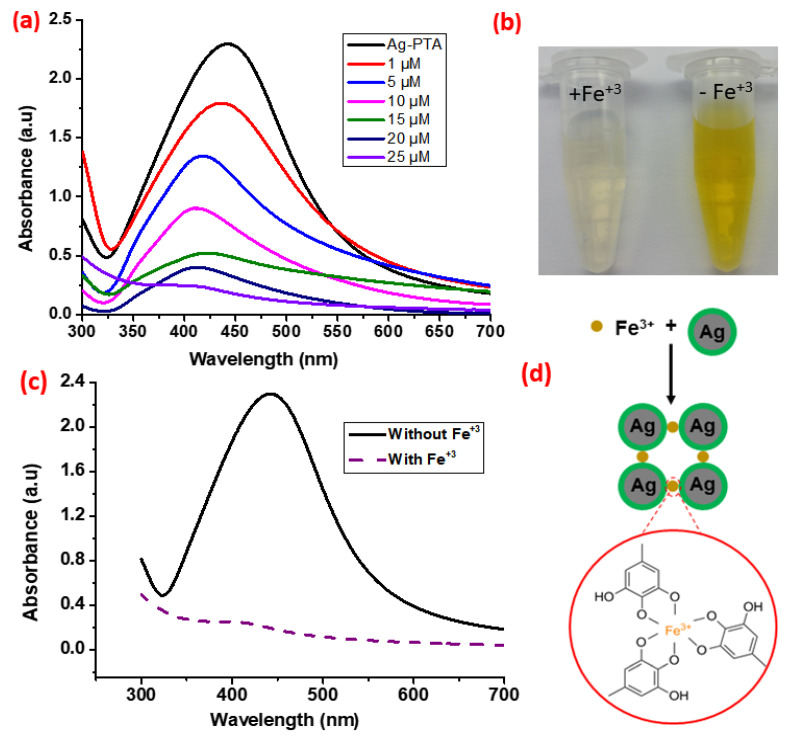
The assay for visual detection of Fe^3+^ ions: (**a**) UV–vis spectra obtained from solutions of synthesized silver nanocomposites; (**b**) photographs of the solution containing functionalized silver nanocomposites (right) and the same solution after the addition of Fe^3+^ ions (25 μM; left); (**c**) UV–vis spectra of aqueous dispersion of synthesized silver composites alone and after 03 min in the presence of Fe^3+^ ions. Solid line: silver nanocomposites without Fe^3+^, dotted line: silver nanocomposites with Fe^3+^ ions; (**d**) coordination of Fe^3+^ ions with poly(tannic acid) shell of the synthesized silver nanocomposites.

**Figure 10:**
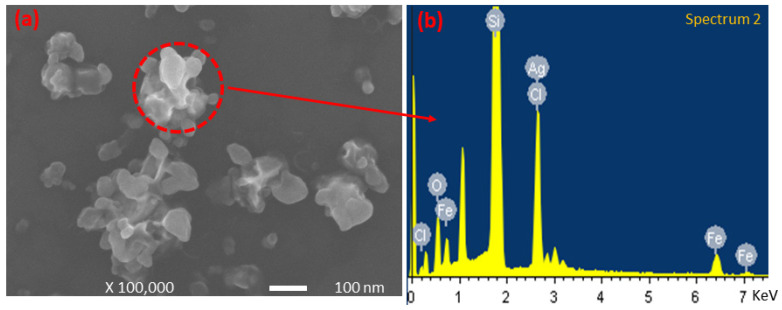
(**a**) SEM image of silver nanocomposite’s aggregate after the addition of Fe^3+^ ions; and (**b**) corresponding EDS analysis of aggregate.

The synthesized silver nanocomposites were used as a sensor for the visual detection of Fe^3+^ ions. This detection method depends upon the aggregation of synthesized silver nanocomposites in an aqueous solution. Fe^3+^ ions coordinate with the PTA present on the surface of the nanocomposites and form aggregates. Upon the addition of various concentrations of ferric ions into the solution under ambient conditions, the yellow color of the solution starts to turn transparent (Figure 9a). The intensity of the characteristic UV–vis absorbance peak (441 nm) of the dispersed nanocomposites gradually reduces with an increasing concentration of ferric ions (Figure 9b). This aggregation phenomenon of the silver nanocomposites in the presence of Fe^3+^ ions can also be observed by the naked eye, which eliminates the need for a specific instrument to detect Fe^3+^ ions. The minimum aqueous concentration of Fe^3+^ ions required for the color change visible to the naked eye was also recorded and analyzed. For this, Fe^3+^ ions were added in the solution of silver nanocomposites at the final concentration of Fe^3+^ ions ranging from 1 to 25 μM (Figure 9a). After 3 min of reaction time, the obvious color change was observed when Fe^3+^ ions ≥ 20 μM, whereas no obvious color change was observed when the ferric ion concentration was <5 μM. To the best of our knowledge, the synthesized silver nanocomposite is the best sensor for the visual detection of ferric ions with the lowest visual detection limit (20 μM) compared to the previously reported results indicating the visual detection limit of 30 [52], 40 [10], 50 [53], and 100 μM [54]. Furthermore, the reported research for the visual detection of ferric ions is either complex, time consuming, or less sensitive [10,52,53,54]. The coordination chemistry of ferric ions with the PTA shells of the nanocomposites is illustrated in Figure 9d. The aggregate formation was also confirmed by the SEM images (Figure 10a), and EDS elemental analysis (Figure 10b) confirmed the coordination of the Fe^3+^ ions with nanocomposites. For the clarity and ease of understanding, the results for the EDS elemental analysis are also summarized in Table 7. A strong signal for Si was observed during the EDS analysis from the silicon wafer that was used as a substrate for the sample preparation.

**Figure 11:**
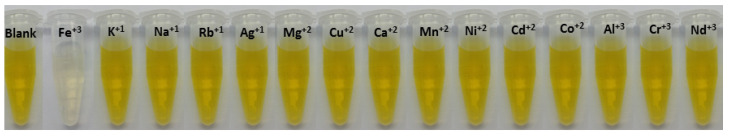
Photograph of the fresh solutions containing mixtures of functionalized Ag-PTA nanocomposites with different metal cations. The ion concentrations of Na^+^, K^+^, Rb^+^, Ag^+^, Mg^2+^, Cu^2+^, Ca^2+^, Mn^2+^, Ni^2+^, Cd^2+^, Co^2+^, Al^3+^, Cr^3+^, and Nd^3+^ were each 100 μM. The concentration of Fe^3+^ was 20 μM.

We also evaluated the high selectivity of silver nanocomposites for Fe^3+^ ions using other monovalent, divalent, or trivalent ions instead of Fe^3+^ ions, including K^1+^, Na^1+^, Rb^1+^, Ag^1+^, Mg^2+^, Cu^2+^, Mn^2+^, Ni^2+^, Cd^2+^, Co^2+^, Al^3+^, Cr^3+^, and Nd^3+^, at a final concentration of 20 μM and found that the synthesized silver nanocomposites were highly selective toward Fe^3+^ ions (Figure 11). There was no visible color change in the nanocomposites even at the 100 μM concentration of ions. No visible color change or aggregation could be observed (except for silver ions) even after five (5) weeks, showing the long-term aqueous stability of the silver nanocomposites-based detection system. Thus, this is the first very simple method based on silver nanocomposites (Ag-PTA) that can be conveniently used for the visual detection of ferric ions with high sensitivity and selectivity.

## 4. Conclusions

In this work, we developed a low-cost, highly selective, and sensitive sensor for the visual detection of Fe^3+^ ions based on silver nanocomposites. Silver nanocomposites were prepared by a simple, low-cost, and one-pot synthesis method by mixing tannic acid with silver salt at room temperature. Tannic acid acts as both a reducing and stabilizing agent. It first reduced the silver nitrate, then underwent a self-oxidation process and formed a thick capping layer of poly(tannic acid). The analyzed results confirmed the successful synthesis of very fine and spherical silver nanocomposites with a thick layer of tannic acid. Relying on the coordination chemistry of galloyl groups of poly(tannic acid), a highly efficient and selective assay for the detection of Fe^3+^ ions was developed. The synthesized silver nanocomposites displayed the visual detection of Fe^3+^ ions with a minimum detection limit of 20 μM for naked eye detection. The highly sensitive visual detection phenomenon can be attributed to the aggregate formation of silver nanocomposites in the presence of Fe^3+^ ions. Additionally, fabric samples drop-coated with synthesized silver nanocomposites exhibited antibacterial response against *E. coli* and *S. aureus*. The inhibition of zones against the microbial burden ranged from 4.6 mm for *S. aureus* to 3.2 mm for *E. coli*. The treated silver samples also indicated notable antifungal activity. Moreover, silver nanocomposites showed that antiviral activity against influenza virus and feline calicivirus reduced both virus titers by 95%, while the untreated sample caused no decline in viral titers. Since iron and its ions are important biological minerals, it is very important that a highly sensitive, selective, easily readable assay is available for the visual detection of Fe^3+^ ions. In this work, we synthesized silver nanocomposites with a thick stabilizing layer of poly(tannic acid) that fulfills these conditions. Based on their unique structure, synthesized silver nanocomposites may find unique futuristic applications, especially in the fields of catalysis, medical, and metal detection.

## Figures and Tables

**Table 1:** Type of material and testing conditions.

Host cell	The Crandell–Rees feline kidney (CRFK) cell ATCC CCL-94	Madin–Darby canine kidney (MDCK) cell ATCC CCL-34
Virus strain	Influenza A virus (H3N2) AATCC VR-1679	Feline calicivirus (FCV) F-9 strain AATCC VR-782
Growth medium	EMEM	RPMI 1640
Maintenance medium	Grade 3 water (800 mL), kanamycin sulphate (60 mg) Eagle’s minimum essential medium (9.53 g)	
No. of washes of sample	20 cycles	
Wash standard	Normal wash ISO 6330-2012	
Washing temperature	40 °C	
Drying standard	Tumbled dry	
Contact time	2 h	

**Table 2:** Types of pathogens used in the study.

Bacteria	Virus	Yeast
Staphylococcus aureus	Feline calicivirus	Candida albicans
Escherichia coli	Influenza virus	

**Table 3:** Detailed summary of the elemental composition of silver nanocomposites.

Name	Peak Binding Energy (eV)	Height of Counts per Second (CPS)	Peak Area (CPS.eV)	Atomic %
Ag3d	368.37	43,720.62	229,982	5.85
O1s	532.79	33,415.45	125,626.3	26.79
C1s	285.41	24,781.04	102,895.8	55.7
N1s	401.9	5227.99	34,148.99	11.66

**Table 4:** Summary of the FTIR analysis of silver nanocomposites.

Sr. No	Frequency (cm^−1^)	Functional Group	Sr. No	Frequency (cm^−1^)	Functional Group
1	3400	Carboxylic acid, phenols	2	2932	C–H asymmetric stretching
3	2841	C–H symmetric stretching	4	1582	Carboxylic acid
5	1415	C–O/C–H bending	6	1220	Ether
7	1096	C–O stretching of hydroxyl group	8	500–850	Aromatic rings

**Table 5:** Reduction percentage of fungal activity.

Sample	Amount of Loaded Silver Nanocomposites (mm)	Reduction %age
1	0	0
2	0.5	9.23
3	0.75	22.34
4	1	46.63
5	1.25	66.45
6	1.5	76.67

**Table 6:** Measurement of virus infectivity in reduction percentage.

Name of Virus	Sample	Toxicity Titer (TCID_50_ per Carrier)	Mean Titer (TCID_50_ per Carrier)	Mean Log _10_ (TCID_50_ per Carrier)	Log _10_ Reduction	Reduction %
Feline calicivirus	Control	5.5 × 10^5^	5.43 × 10^5^	5.73	2.19	99.45%
		4.5 × 10^5^				
		6.3 × 10^5^				
	Test sample	3.8 × 10^5^	3.5 × 10^3^	3.54		
		2.4 × 10^5^				
		4.3 × 10^5^				
Influenza virus	Control	6.7 × 10^5^	5.53 × 10^5^	5.74	2.3	99.52%
		4.5 × 10^5^				
		5.4 × 10^5^				
	Test sample	2.1 × 10^5^	2.8 × 10^3^	3.44		
		4.4 × 10^5^				
		1.9 × 10^5^				

**Table 7:** Summary of the results for the EDS analysis of the aggregate of silver nanocomposites.

Sr. No	Element	Weight %	Atomic %
1	O K	9.84	17.43
3	Si K	68.95	69.61
5	Cl K	11.01	8.81
7	Fe K	6.01	3.05
8	Ag L	4.20	1.10
	Total	100	

## Data Availability

Not applicable.
